# Ultrasonic Wave Mode-Based Application for Contactless Density Measurement of Highly Aerated Batters

**DOI:** 10.3390/foods12091927

**Published:** 2023-05-08

**Authors:** Michael Metzenmacher, Dominik Geier, Thomas Becker

**Affiliations:** Chair of Brewing and Beverage Technology, TUM School of Life Sciences, Technical University of Munich, 85354 Freising, Germany; michael.metzenmacher@tum.de (M.M.); tb@tum.de (T.B.)

**Keywords:** ultrasonic measurement, density measurement, batter structure, aerated batter, continuous process, mode conversion

## Abstract

An ultrasonic wave mode-based method for density measurement in highly foamed batters was developed. Therefore, a non-contact ultrasonic sensor system was designed to generate signals for batch-wise processes. An ultrasonic sensor, containing a piezoelectric ceramic at the fundamental longitudinal frequency of 2 MHz, was used to take impedance measurements in pulse-echo mode. The ultrasonic signals were processed and analysed wave-mode wise, using a feature-driven approach. The measurements were carried out for different mixing times within a container, with the attached ultrasonic sensor. Within the biscuit batter, the change to the ultrasonic signals caused by density changes during the batter-mixing process was monitored (R^2^ = 0.96). The density range detected by the sensor ranges between 500 g/L and 1000 g/L. The ultrasonic sensor system developed also shows a reasonable level of accuracy for the measurements of biscuit batter variations (R^2^ > 0.94). The main benefit of this novel technique, which comprises multiple wave modes for signal features and combines these features with the relevant process parameters, leads to a more robust system as regards to multiple interference factors.

## 1. Introduction

The quality of baked products depends on many factors. To ensure consistently high quality, the production process must be capable of adapting to the fluctuating qualities of the raw materials [1]. Even the short-term storage of flour causes changes to its baking characteristics [2]. Quality-related attributes, such as the specific volume of cake or the porous structure of crumbs, depend on both the cake batter recipe and the general baking conditions [3,4,5].

For highly foamed baked goods, such as biscuits, the air content in the batter affects the texture and quality of the final product significantly. The structure of the final product, however, depends heavily on the process used to mix the cake batter, whereby this constitutes one of the major processes in any bakery system. During mixing, air is introduced during a liquid phase, which consists of egg, sugar, wheat flour and other ingredients, to form the batter. As there is no additional raising agent, the final structure of sponge-cake depends on the air trapped within the continuous phase [6,7] and is, therefore, related to its density [8]. During mixing, the density changes from 1000 g/L (poorly aerated) to under 600 g/L (highly aerated). The mixing time thus depends on the density. This is because of the amount of air bubbles introduced into the batter during the process increases and, ultimately, ends up affecting the cake volume. Therefore, the density of the batter must be measured [9]. To monitor critical process steps such as mixing, real-time data are required: such data afford a deeper insight into the current state of the process. Thus, online sensors, which are able to take measurements directly within the process as well as inline sensors measuring bypass or required samples, are suitable for this purpose [10].

Within the food industry, there is a high interest in online systems measuring the density and the textural properties of food products. Currently, the usual procedure for density measurement uses a gravimetric offline measurement of the batter. It does this by analysing samples of fixed volumes. Because of this analogue, time-consuming step, there is a need to develop a non-invasive, online method for determining the density in baking processes. 

Another common method used to study the mixing process is based on measuring work input and torque, using a strain gauge. The mixing process is stopped at a predefined total energy input, which is calculated beforehand by integrating the power over time [11,12]. Using this method, however, only non-specific physical modifications can be analysed, while the method itself depends heavily on the type of vessel and the mixture employed. Other direct rheological measurements were performed for different dough systems, such as yeasted bread doughs [13], sour doughs and cake batters. The mixing process can also be investigated by means of X-ray CT (computed tomography) measurements. The detection of bubble size and oxygen dispersion within a dough mix has already been shown [14]. This method has a high level of accuracy but is not suitable for inline measurement, due to the complex evaluation process it necessitates. 

Ultrasonic-based sensors are perfectly suited to measurement systems operated during food processing, due to their hygienic, non-invasive and non-destructive behaviour [15,16,17]. The use of ultrasonic-based measurements for solid dough compounds is already well-established [18]. It is already possible to measure wheat quality [19] and the precise composition of wheat-flour–water doughs [20] using ultrasonic methods. The influence of the properties of bread dough during mixing [20,21,22] and fermentation [23,24] as detected by ultrasonic signals has been shown. Recently, it was also shown that it is possible to monitor the mixing process for liquid model systems using ultrasound [25]. It is even possible to detect air bubbles in dough with ultrasonic methods [26,27]. The impact of air within a more liquid-like product seems to be more challenging, however. There are only a few reports of ultrasonic-based investigations of batters [28]. Because of the higher air content in batters compared to dough systems, ultrasonic signals are dampened almost completely within the medium and are therefore not suitable for transmission. Hence, only ultrasonic measurement methods that do not depend on acoustic transparency can be used.

Ultrasonic waves for measurement systems are generated mostly by piezo-ceramic transducers, which convert electrical energy into mechanical energy and vice versa. The measurement method can be divided up into three basic components: reflection, transmission and emission. 

Reflection measurements, using either one (pulse-echo mode) or multiple transducers (pitch-catch mode), send and receive the reflected wave at a given interface. Transmission measurements are realized with a sending and receiving transducer, where the ultrasonic signal is transmitted through the material, whereas for emission measurements, the sound signals recorded are emitted by the process itself. Ultrasonic waves for measurement applications are mostly low power and high frequency, in order to prevent changes to the media they are transmitted through [15]. In the reflection mode, impedance, as measured at the surface of the batter [9], correlates with density. 

Other studies have also shown the feasibility of ultrasonic techniques for density measurement in batter [9,29,30,31] but only for poorly aerated batters (~1000 g/L). For highly aerated batters, however, the high reflection and distortion rates of ultrasonic waves in gas bubbles do not allow transmission measurements. Therefore, only reflected, scattered waves can be used for analysis purposes. Furthermore, the ultrasonic waves can be influenced by reflection and scattering at each interface used. The resulting ultrasonic signals interact at all of the interfaces individually and are then subjected to mode conversion [32]. Mode conversion occurs when an ultrasonic wave faces an interface between materials of different impedances and the incident angle to the interface is abnormal. Some of the sound energy introduced is therefore reflected at the interface between the buffer rod and the surface of the batter, due to the acoustic impedance. The acoustic impedance *Z* can be described with Formula (1).

(1) $$Z=\frac{p}{u}=\rho c$$

p: sound pressure, p: sound particle velocity, 𝜌: density, c: ultrasonic velocity, Z: impedance.

Due to the different impedances of the two different media (buffer rod—batter), reflections occur at their mutual interface. The reflection coefficient R can be described with Formula (2).

(2) $$R=\frac{p_{d}}{p_{e}}=\frac{Z_{2}-Z_{1}}{Z_{2}+Z_{1}}$$

$Z_{1}$: acoustic impedance of medium 1, $Z_{2}$: acoustic impedance of medium 2, $p_{e}$: emitted sound energy, $p_{d}$: detected sound energy, R: reflection coefficient.

The ratio between the emitted sound energy *p*_e_ and detected sound energy *p*_d_ depends on the acoustic properties of both interfaces, as represented by the acoustic impedance *Z*_1_ of the medium M_1_ and *Z*_2_ of the medium M_2_. Due to the differing impedances of the phases (solid/liquid/air), a mode conversion also occurs between longitudinal, shear and surface–acoustic waves [33,34]. At the changing interfaces between the batter structure and the air bubbles within the batter, the original ultrasonic waves scatter and meet the air bubbles at different angles. Due to the characteristic of batter being a non-Newtonian fluid (i.e., neither solid nor liquid), the ultrasonic waves are also mode converted within the batter (longitudinal to shear waves) [35].

As regards the interpretation of ultrasonic signals, the use of special analytical strategies plays a decisive role. There are two main kinds of approaches to predicting the desired process parameters by means of ultrasonic signal data: model-based approaches and machine-learning approaches. 

Model-based approaches rely on expert knowledge of the processes and the underlying physical relations. Gaining expert knowledge of the whole system is a very complicated undertaking, while, ultimately, due to their inherent complexity, both the process and the production elements here are affected by a large number of factors [36]. 

On the other hand, the machine-learning approaches do not require expert knowledge but rather, a considerable amount of sensor data, in order to create a decent model. For modelling complex processes, using only expert knowledge-based approaches is very difficult and, therefore, not possible for many process steps. The downside of the machine-learning approach using deep learning algorithms is that a large amount of data is required in order to come up with a proper prediction. The ultrasonic features of the approach used in this study are based on the physical behaviour of the biscuit masses. Thus, expert knowledge can be integrated into the machine-learning approach via ultrasonic features.

This work shows an ultrasonic-based, non-invasive low-cost method for determining the density of highly aerated batter during a batch process. The method therefore uses a feature analysis, which is based on impedance measurement regarding mode conversion, to correlate ultrasonic signals with batter density. This also applies to different recipes (e.g., standard, gluten free).

## 2. Materials and Methods

### 2.1. Materials

Wheat flour (Type 550) was supplied by Rosenmehl, Rosenmühle GmbH (Ergolding, Germany). Pasteurized whole egg, white sugar and wheat starch were purchased from the local market. The emulsifier (Colco) was supplied by Aromatic (Aromatic Marketing GmbH—Berlin, Germany).

### 2.2. Batter Formulation

A standard procedure based on industrial production was used for the batter formulation. A set of four batters was prepared with different sugar, flour, wheat starch and emulator contents (see Table 1). The standard mixture was varied (with/without starch/emulsifier) in order to obtain both different batter properties (standard and gluten free) and thermally fixed cakes with varied physical characteristics as a result of varying aeration. All of the ingredients, which were tempered to room temperature, were pre-mixed at a low speed for 120 s. The foaming process was started afterwards by high-speed mixing for various durations, using a KitchenAid Professional mixer 5KPM5 (KitchenAid, Benton Harbor, MI, USA). The temperature measured after the completion of the foaming process was 22 ± 2 °C.

### 2.3. Density Measurement

The batter was characterized after the completion of the foaming process. The measurements were performed at a weight of 1 L (CSM Deutschland GmbH, Bingen, Germany), with 100 cm^3^ at a temperature of 22 ± 2 °C. The cup was filled with batter and levelled. The weight was determined using a laboratory balance (Kern, accuracy to ±0.5 mg). To determine the density, the average of three samples was taken and calculated, using the ratio of the mass of batter and the volume of the litre weight. The density can be described using Formula (3).

(3) $$\rho =\frac{m_{liter\;weight+batter}-m_{liter\;weight}}{100\;{cm}^{3}}*10$$

$m_{liter\;weight}$: mass of empty litre weight [g]; $m_{litre\;weight+batter}$: filled litre weight with batter [g]; ρ: density of batter [g/L]

### 2.4. Ultrasonic Measurement System

The ultrasonic-based measuring system developed consists of a microcontroller for signal generation, as well as a signal recording element. The data obtained from the ultrasonic transducer was stored and evaluated, using Virtual Expert 4.0 software (Gimbio GmbH, Freising, Germany). The self-developed round-shaped ultrasonic transducers with a diameter of 12 mm, which are based on a piezoceramic (PIC 255, PI Ceramic GmbH, Lederhose, Germany), used a median frequency of 2 MHz and were encapsulated in resin and tungsten-based backing material. This was to avoid ringing and to guarantee an adequate signal resolution. The piezo transducer was excited by a rectangular 100 V excitation of 250 ns, with three consecutive pulses, in order to obtain stable signals. 

**Figure 1:**
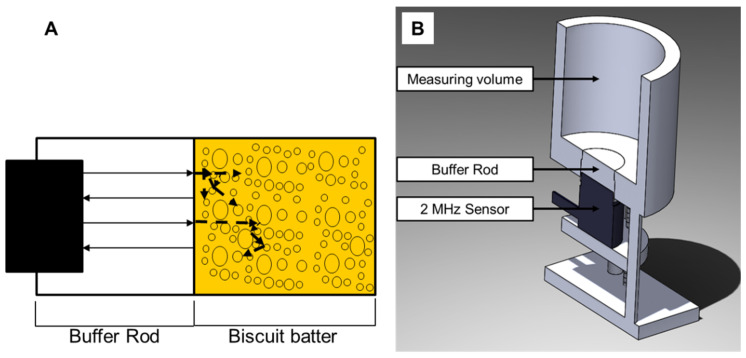
Schematic representation of the ultrasonic-based measuring system. (**A**) The ultrasonic waves are propagating through the buffer rod and are reflected at the surface between buffer-rod/batter and batter/bubbles. (**B**) Sectional view of the measurement set-up.

Because of the heavily whipped batters, it was difficult to obtain the transmission measurements. Therefore, the measurement principle was based on the pulse–echo method. Thus, with the pulse–echo measurement method used, the exiting transducer and the receiving transducer were the same. Directly after sending the electrical impulse to the transducer, the set-up switched from send to record. The ultrasonic signals returning were recorded for 60 µs. To avoid the product contact and to guarantee measurements in the far field, the ultrasonic signals were introduced into the medium by an ultrasonic transducer via a polymethyl methacrylate (PMMA) buffer rod (see Figure 1A). Because the pressure wave originated from several points along the piezo transducer disc, the waves interfered in the region directly in front of the sensor (near field). Therefore, the pressure field was not constant up to a certain distance (far field). The natural focus (transition between the near field and far field) can be calculated using Formula (4) [37].

(4) $$N\approx \frac{D^{2}}{4\lambda }=\frac{A}{\pi \lambda }$$

*N*: natural focus length (mm), *D*: ultrasonic transducer diameter (mm), *A*: sensor area (mm^2^), *λ*: wavelength of ultrasonic wave propagation in the medium (mm).

The wavelength can be calculated using Formula (5) [37].

(5) $$\lambda =\frac{c}{f}$$

*c*: wave velocity of ultrasonic wave propagation in the medium (m/s), *f*: ultrasonic frequency (Hz).

For PMMA at room temperature, the ultrasonic wave velocity for 2 MHz is approximately 2740 m/s [38]. The far field calculated for a 12 mm piezo disc sensor for PMMA was thus 26.28 mm. The buffer rod therefore had a length of 30 mm, in order to ensure a measurement in the ultrasonic far-field. The 2 MHz ultrasonic sensor was attached at the bottom of the measurement chamber, which could be filled easily from above (see Figure 1B). 

The acoustic properties of batter are subject to change. This is due to batter whipping whenever air is introduced and to the consequent effect on the reflection coefficient R (see Formula (2)). The signal energy, which penetrates the batter, is reflected and scattered at gas bubbles and other surfaces of the batter, as well as mode converted. Therefore, considering the reflection coefficient alone is not enough to determine the density of highly aerated batters.

### 2.5. Signal Pre-Processing

**Figure 2:**
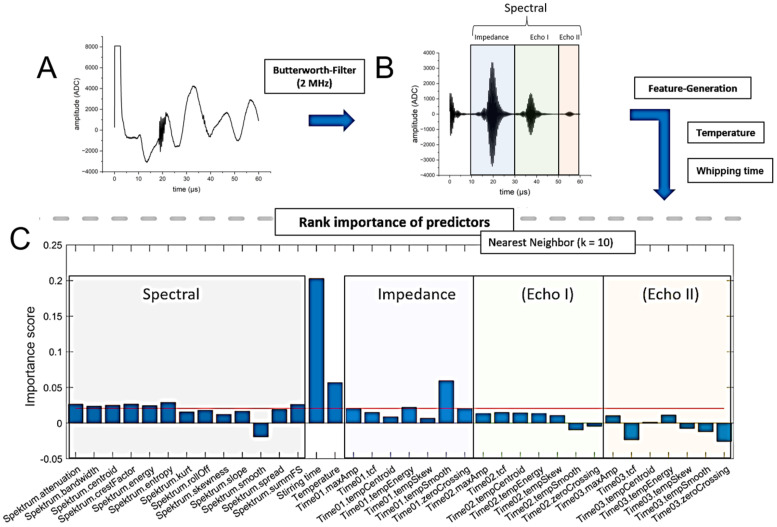
Schematic representation of the ultrasonic signal for evaluation by means of feature analysis. (**A**) Ultrasonic raw signal, (**B**) filtered ultrasonic signal divided into the features areas, (**C**) rank of importance of the features and selection of highest importance weighting features (above red line).

To eliminate noise interference, e.g., due to pumps or motors, the ultrasonic signals were analysed and filtered by an 8th order Butterworth band pass filter with band threshes at 1.75 and 2.25 MHz (see Figure 2A,B). The signals were collected using Virtual Expert 4.0 software at a sampling rate of 50 MHz for 60 µs. For analysis, the signal-processing toolbox within MATLAB (Version 2018a) was used. Due to the pulse–echo measurement, the signal could be divided into four sections in the time domain (see Figure 2B):

1. Sensor ringing (0–10 µs)
2. Echo of impedance signal (PMMA/batter) (10–30 µs)
3. First echo of reflections and scattering effects in batter (30–50 µs)
4. Second echo of reflections and scattering effects in batter (50–60 µs)

The sensor ringing in the first section was independent from the actual measurement and thus was not considered. The remaining time sections (Section 1, Section 2 and Section 3) were analysed subsequently via ultrasonic features. Since frequency shifts can occur in ultrasonic-based mode conversion, as well as with scattering and reflection, the ultrasonic signal was also analysed in the frequency domain. Therefore, the signal was converted by means of a discrete Fourier transformation [39] (Formula (6)).

(6) $$X_{k}=\sum_{n=0}^{N-1}x_{n}e^{-\frac{2\pi i}{N}kn},k=0,\ldots ,N-1$$

The Fourier transformation was calculated for the combined sections of the time domain signal and used subsequently for the frequency domain features. 

### 2.6. Ultrasonic Feature Creation

Suitable signal parameters describing the density change were generated from the pre-processed signal data. Physically related parameters, such as acoustic impedance or signal energy, as well as non-specific parameters from the time and frequency domains, were considered to predict the density changes. Because of the major temperature dependencies of ultrasonic propagation, the process temperature was monitored and included in the analysis. The stirring time as a physically based variable was also included as a feature. The following features were adapted from the field “speech, music and environmental sounds” [40] and used for the analysis.

#### 2.6.1. Time Domain Features

*MaxAmp* shows the highest amplitude value of the signal (Formula (7)).

*maxAmp:*

(7) $$maxAmp=max\left(\left|x\left(n\right)\right|\right)$$

The temporal crest factor (tcf) describes the ratio between the maximum amplitude and the average amplitude and represents the singularity of the signal (Formula (8)).

*tcf (Temporal crest factor)* *:*

(8) $$tcf=\frac{max\left(\left|x\left(n\right)\right|\right)}{\frac{1}{N}\sum_{n=1}^{N}\left|x\left(n\right)\right|}$$

Temporal centroid is the time at which half the energy of the signal is reached. It is not necessary for this time to be at the zero point (Formula (9)).

*tempCentroid:*

(9) $$C_{t}=\frac{\sum_{n=1}^{N}n\cdot {\left(x\left(n\right)\right)}^{2}}{\sum_{n=1}^{N}{\left(x\left(n\right)\right)}^{2}}$$

Temporal energy shows the energy of the whole signal, described by the area under the signal curve by summing up the absolute values of the signal (Formula (10)).

*tempEnergy:*

(10) $$tE=\sum_{n=1}^{N}{\left(x\left(n\right)\right)}^{2}$$

Temporal skewness is a measure of the asymmetry of signal amplitudes, based on the normal distribution (Formula (11)).

*tempSkew:*

(11) $$m_{3}=\sum_{n=1}^{N}{\left(n-C_{t}\right)}^{3}\cdot \frac{\left|x\left(n\right)\right|}{\sum_{n=1}^{N}\left|x\left(n\right)\right|}\;tsp=\sum_{n=r1}^{r2}{\left(n-C_{t}\right)}^{2}\cdot \frac{\left|x\left(n\right)\right|}{\sum_{n=1}^{N}\left|x\left(n\right)\right|}\;Ts=\frac{m_{3}}{{tsp}^{3/2}}$$

Temporal smoothness shows the variation of the signal amplitude related to the direct neighbours of a signal point (Formula (12)). 

*tempSmooth:*

(12) $$tmpSm=20\cdot \sum_{n=2}^{N-1}\left|log\left|x\left(n\right)\right|-\frac{log\left|x\left(n-1\right)\right|+log\left|x\left(n\right)\right|+log\left|x\left(n+1\right)\right|}{3}\right|$$

The zero crossing rate is the number of points at which the amplitude crosses the zero point (Formula (13)).

*zeroCrossing:*

(13) $$zCros=\frac{1}{2\cdot N}\sum_{n=2}^{N}\left|sign\left(x\left(n\right)\right)-sign\left(x\left(n-1\right)\right)\right|\;\left\{\begin{array}{l} sign=1;x\left(n\right)>0\\ sign=0;x\left(n\right)=0\\ sign=-1;x\left(n\right)<0 \end{array}\right.$$

#### 2.6.2. Spectral Domain Features

Spectral attenuation shows the energy of the entire frequency spectrum, as described by the area under the spectrum curve by summing the values of the signals and the subsequent logarithmic conversion (Formula (14)).

*at* *tenuation:*

(14) $$att=10\cdot \log_{10}\sum_{m=1}^{N}\left|X(m)\right|²$$

The bandwidth shows the bandwidth around the main frequency that is above a certain threshold value (Formula (15)). 

*bandwidth* *:*

(15) $$bw=\Delta f=f_{2}-f_{1}$$

The spectral centroid is the frequency at which half the energy of the signal is reached (Formula (16)). 

*centroid* *:*

(16) $$C_{s}=\frac{\sum_{n=1}^{N}n\cdot {\left(x\left(n\right)\right)}^{2}}{\sum_{n=1}^{N}{\left(x\left(n\right)\right)}^{2}}$$

The spectral crest factor describes the ratio between the maximum frequency and the average frequency and represents the singularity of the frequencies (Formula (17)). 

*crestFactor* *:*

(17) $$Crestf=\frac{max\left(\left|x\left(n\right)\right|\right)}{\frac{1}{N}\sum_{n=1}^{N}\left|x\left(n\right)\right|}$$

The spectral energy shows the energy of the whole frequencies, as described by the area under the frequency curve by summing up the values of the Fourier-transformed signal (Formula (18)). 

*energy* *:*

(18) $$Energy=\sum_{n=1}^{N}{\left(x\left(n\right)\right)}^{2}$$

The spectral entropy shows the total loss of frequency energy and calculates the loss by summing the energy of the frequencies point by point (Formula (19)). 

*entropy* *:*

(19) $$Entropy=-\sum_{n=1}^{N}{\left(\frac{\left|x\left(n\right)\right|}{\sum_{n=1}^{N}\left|x\left(n\right)\right|}\right)}^{2}ln{\left(\frac{\left|x\left(n\right)\right|}{\sum_{n=1}^{N}\left|x\left(n\right)\right|}\right)}^{2}$$

The spectral kurtosis is a measure of the flatness (slope) and the singular variation of the frequency spectrum around the frequency maximum (Formula (20)). 

*k* *urtosis* *:*

(20) $$Kurt=\frac{\sum_{m=1}^{N}{\left(m-C_{t}\right)}^{4}\cdot {\left(\frac{\left|x\left(m\right)\right|}{\sum_{m=1}^{N}\left|x\left(m\right)\right|}\right)}^{2}}{{\sum_{m=1}^{N}{\left(m-C_{t}\right)}^{2}\cdot {\left(\frac{\left|x\left(m\right)\right|}{\sum_{m=1}^{N}\left|x\left(m\right)\right|}\right)}^{2}}^{2}}\left\{\begin{array}{c} <3\;flatter\;distribution\\ =3\;normal\;distribution\\ >3\;peaker\;distribution \end{array}\right.$$

The rollOff shows the frequency at which 90% of the energy of the frequencies has already been received (Formula (21)). 

*r* *ollOff* *:*

(21) $$rollOff=\sum_{m=1}^{m_{rf}}\left|X\left(m\right)\right|=0.9\cdot \sum_{m=1}^{1024}\left|X\left(m\right)\right|$$

The spectral skewness is a measure of the asymmetry of the frequency pattern, based on the normal distribution (Formula (22)). 

*Skewness* *(Ts)* *:*

(22) $$m_{3}=\sum_{n=1}^{N}{\left(n-C_{t}\right)}^{3}\cdot \frac{\left|x\left(n\right)\right|}{\sum_{n=1}^{N}\left|x\left(n\right)\right|}\;tsp=\sum_{n=r1}^{r2}{\left(n-C_{t}\right)}^{2}\cdot \frac{\left|x\left(n\right)\right|}{\sum_{n=1}^{N}\left|x\left(n\right)\right|}\;Ts=\frac{m_{3}}{{tsp}^{3/2}}$$

The spectral slope shows the decrease in the frequency energy over the frequencies by linear regression (Formula (23)). 

*slope* *:*

(23) $$Slope=\frac{N\cdot \sum_{m=1}^{N}\left(m\cdot \left|X\left(m\right)\right|\right)-\sum_{m=1}^{N}m\cdot \sum_{m=1}^{N}\left|X\left(m\right)\right|}{\sum_{m=1}^{N}\left|X\left(m\right)\right|\cdot \left(\sum_{m=1}^{N}m^{2}-{\left(\sum_{m=1}^{N}m\right)}^{2}\right)}$$

The spectral smoothness shows the variation of the frequency power with respect to the direct neighbours of the main frequency (Formula (24)). 

*smooth* *:*

(24) $$smt=20\cdot \sum_{n=2}^{N-1}\left|log\left|x\left(n\right)\right|-\frac{log\left|x\left(n-1\right)\right|+log\left|x\left(n\right)\right|+log\left|x\left(n+1\right)\right|}{3}\right|$$

The spectral spread shows the variance of the main frequency energy around the mean value of the total frequency energy (Formula (25)). 

*s* *pread* *:*

(25) $$ssp=\sum_{m=1}^{N}{\left(m-C_{s}\right)}^{2}\cdot \frac{\left|X\left(m\right)\right|}{\sum_{m=1}^{N}\left|X\left(m\right)\right|}$$

The spectral summFS shows the energy of a frequency band defined by two boundaries, described by the area under the signal curve by summing its values (Formula (26)).

*s* *ummFS* *:*

(26) $$summFs=\sum_{n=1}^{N}{\left(x\left(n\right)\right)}^{2}$$

### 2.7. Feature Selection and Regression Method

In a first step, all ultrasonic features presented previously were generated for each ultrasonic signal (see Figure 2A). To enhance the informative value of each signal, the features were taken for different time-areas (impedance, Echo I, Echo II). The time-areas therefore can be divided into echo parts and an impedance part (see Figure 2B). The spectral features were computed for the whole ultrasonic signal. Additionally, the stirring time and the temperature measured were used as features too. To avoid overfitting, the importance ranking of the predictors was applied using the nearest-neighbour method [41] based on the ten nearest neighbours. For further analysis, only the ten features (above the red line) with the highest importance weighting were kept (see Figure 2C).

To regress the features generated to the density of the batter, a Gaussian process regression (GPR) was performed [42]. The Gaussian process regression is a non-parametric kernel-based probabilistic model, which permits modelling data with varying multiple scales. The rational GPR model used can be described with Formula (27).

(27) $$k\left(x_{i},x_{j}|\theta \right)=\sigma_{f}^{2}(1+\frac{r^{2}}{2\alpha \sigma_{i}^{2}})\;\mathrm{where}:\;r=\sqrt{{\left(x_{i}-x_{j}\right)}^{T}(x_{i}-x_{j})}$$

θ: maximum of posteriori estimates, $\sigma_{f}$: signal standard deviation, α: non-negative parameter of the covariance.

To select and train the GPR model, 70% of the measured data were used; 30% of the data were used for validation.

## 3. Results and Discussion

### 3.1. Density Measurement for Standard Batter without Variation

**Figure 3:**
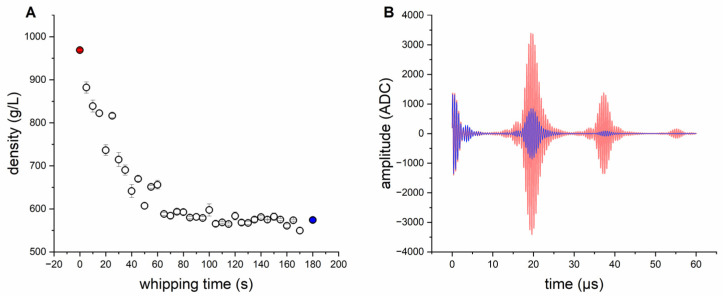
(**A**) Time profile of density as a function of whipping time for the batch process of standard biscuit batter from 0 s (red) to 180 s (blue). (**B**) Ultrasonic signal change between 0 s (red) and 180 s (blue) whipping time.

**Figure 4:**
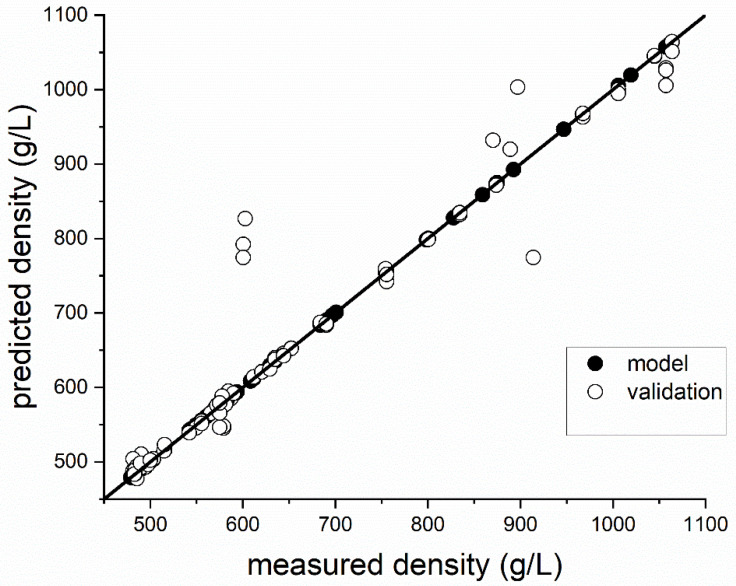
Relationship between density predicted by ultrasonic signals and the reference (gravimetric determination) for standard I batter; model (dot (●), R² = 0.99, RMSE = 0.03), validation (circle (○), R² = 0.91, RMSE = 54.69).

A first experiment was performed, in order to test the ultrasonic sensor with differently aerated standard biscuit batter. All tests were performed in three-fold determination. The time profile of density, as a function of whipping time for standard biscuit batter (see Table 1), is shown in Figure 3A. During mixing at high speed, the density of the standard batter drops quickly and remains steady after about 100 s. Longer aeration (more than 180 s) showed a negligible change on the extended aeration and density loss. Due to the foam-stabilizing effect of emulsifiers, only the foam structure changes [43] after 180 s of whipping time, whereas the density remains constant. The changes in the ultrasonic signal between 0 s and 180 s is shown in Figure 3B. The signal deflection in the signal from the ultrasonic sensor in the range between 0 and 10 µs is due to ringing of the sensor; and it is, therefore, it is not conducive to the measurement of material properties. Changes in the signal can mainly be detected because of the impedance signal (reflection PMMA/batter, 10–30 µs), although barely for the first echo (30–50 µs). The second echo (>50 µs) can only be detected with poorly aerated batters. Due to the strong attenuation and scattering effects of aerated batter for ultrasonic waves, the sound waves probably penetrate the biscuit batter only marginally and, ideally, are reflected back in such a way that these signals reach the ultrasonic receiver again (see Figure 1A). Therefore, the features generated from the echo areas play a minor role (see Figure 2C). At low densities, due to the increase in air bubbles in the batter, the ultrasonic signal is more scattered and therefore dampened. The prevailing longitudinal waves of the ultrasonic transducer are thus reflected at each interface (batter/air) and converted into other waveforms (surface waves, shear waves, etc.). By filtering the ultrasonic signals at the longitudinal frequency (1.80–2.25 MHz), the signal areas of the ultrasonic signals converted disappear from the signals shown. This is due to other frequency ranges and so they can only be detected within a certain frequency range. Therefore, the frequency-based features are also accorded greater importance (see Figure 2C). Utilizing the resulting features for the GPR model (see Formula (27)), a correlation of the density can be realized (see Figure 4). Since the measurement mainly takes place at the boundary surface of the buffer rod via the ultrasonic sensor, sticking batter residues or unfavourable bubble adhesion onto this surface can cause measurement outliers (see Figure 4). Except for these rare erroneous measurements, the density of standard biscuit batter can be measured in total up to a NRMSE (Normalized Root Mean Squared Error) of 6.32% (see Table 2).

### 3.2. Density Measurement for Batter Variations

**Figure 5:**
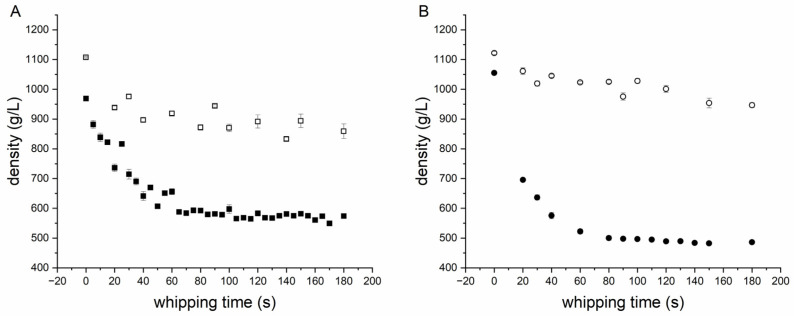
Time profile of density as a function of whipping time for (**A**) standard I batter (black square) and standard II (square) without an emulsifier and (**B**) gluten-free I batter (black dot) and gluten-free II (dot) without an emulsifier.

**Figure 6:**
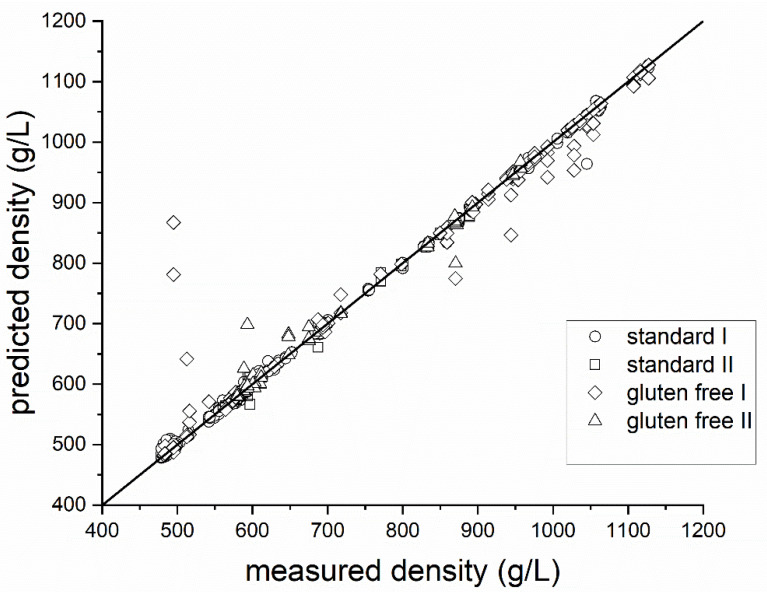
Relationship between density predicted by ultrasonic signals and the reference (gravimetric determination) for all variations (circle: standard I, R² = 0.99, RMSE = 6.32; square: standard II, R² = 0.99, RMSE = 7.46; diamond: gluten-free I, R² = 0.96, RMSE = 42, triangle: gluten-free II, R² = 0.94, RMSE = 18.66).

A second experiment was performed in order to test the ultrasonic sensor with differently aerated standard and gluten-free biscuit batter. Furthermore, variations without an emulsifier were tested, as this has a major impact on the ability of biscuit batters to retain air bubbles. The time profile of density as a function of whipping time for all of the batter variations tested (see Table 1) is shown in Figure 5. The experiment series without an emulsifier could hardly be foamed within the period examined of 180 s. Only with significantly longer whipping times (>10 min), was it possible for the densities of the samples containing an emulsifier to be achieved approximately. The experiments also showed that the required density of approximately 600 g/L for highly aerated batters can be achieved, for both the standard batter and the gluten-free batter, by foaming with emulsifier. The whipping time required to reach this density hardly differs between the standard and gluten-free batter. It turns out that the gluten-free variant can produce lower overall densities. However, this only applies with the use of emulsifiers. Without emulsifiers, the standard biscuit batter whips up faster and can reach lower densities in the same time. This is because gluten itself can serve as an emulsifier, which would stabilize the structure in a standard biscuit. With regard to the features used, this means that the external feature of “whipping time” plays a less significant role when viewed overall. Due to the mode conversion of the ultrasonic signal at every boundary phase, the frequency spectrum of the ultrasonic signal showed changes in the density of the batter. Therefore, seven out of the ten features selected are spectral features (see Figure 2C). When using the same features from the previous experiment for modelling purposes, better results can be achieved overall with the extended dataset (see Figure 6). Outliers can also occur in these series of measurements, due to sticking batter residues or unfavourable bubble adhesion. However, these outliers occur preferentially in gluten-free samples for highly aerated batters due to the lack of stabilization by the gluten networks. The density prediction of the model for standard batter with emulsifier (R^2^ = 0.99, NRMSE = 1.31%) and without emulsifier (R^2^ = 0.99, NRMSE = 1.55%) is therefore slightly better than for gluten-free batter with (R^2^ = 0.96, NRMSE = 8.88%) and without (R^2^ = 0.94, NRMSE = 3.89%). A summary is presented in Table 3. In this work, only two varying biscuit masses (gluten containing, gluten free) without oil and fats were investigated. It was shown that the method also works for this type of variation in the foam system. However, for the determination of the density of other masses (oil containing, etc.) with varying rheological properties, the ultrasonic-based measurement system must be further adapted, since the surface properties change considerably. While in earlier publications the ultrasound-based measurement of density in grain-based foams was only presented as a potential (R^2^ < 0.7) [9,31], the method developed here can be used to make a significantly better prediction (R^2^ > 0.9) of the density, even for highly foamed batters.

## 4. Conclusions

In this paper, the design of an ultrasonic sensor for the mode-conversion measurement via ultrasonic features of highly aerated batters has been described. The ultrasonic sensor system developed was realized as a non-invasive system and, therefore, does not affect the process. The variation of bubbles in the batter due to alternating mixing times changes the density of the batter. In this way, by measuring the mode conversion with respect to the acoustic features of the batter, its density can be measured down to >500 g/L. Although the results already yield a reasonably prediction for highly aerated batters, (NRMSE = 6.32%), including for variated batter formulations (NRMSE = 4.15%), the method can still be extended and optimized.

Future research will extend the pulse–echo approach with multiple sensors for surface acoustic wave measurements in pitch–catch mode. This is in order to improve the accuracy and robustness and to increase the data from the signals due to the ultrasonic mode conversion. In this study, the final ultrasonic model was adjusted to the entire measurement range, as well as to the batter variations. To enhance this model, a restriction to one variation and a specific density range would be beneficial. In addition, for the machine-learning approach used, the rational GPR model was selected, by way of example, in order to provide the proof of concept. By comparing different machine-learning models (support vector regression (SVR), partial least-square regression (PLSR), neural network etc.) and by optimizing them [44], the approach shown in this study could be improved further. Another research direction would be to use the methodology developed in this study to measure further structural parameters (e.g., void fraction) of cereal-based foams [43] or to extend the method for further dough matrices. This would offer [43] a deeper insight into inline measurement systems.

## Figures and Tables

**Table 1:** The composition of the different sponge cake batter variants.

Ingredient	Ingredient Consumption g/5 kg			
	Standard I	Standard II	Gluten-Free I	Gluten-Free II
Whole egg	1417	1417	1417	1417
Wheat flour (Type 550)	750	750	0	0
Wheat starch	925	975	1675	1725
White sugar	1858	1858	1858	1858
Emulsifier	50	0	50	0

**Table 2:** Summary of the R^2^, RMSE (Root Mean Squared Error) and NRMSE (Normalized Root Mean Squared Error) of the model for standard batter.

	R^2^	RMSE	NRMSE [%]
**Model**	0.99	0.03	0.006
**Validation**	0.91	54.69	11.41
**Total**	0.96	30.31	6.32

**Table 3:** Summary of the R^2^, RMSE (Root Mean Squared Error) and NRMSE (Normalized Root Mean Squared Error) of the models for batter variations.

	R^2^	RMSE	NRMSE [%]
**Standard I**	0.99	6.32	1.31
**Standard II**	0.99	7.46	1.55
**Gluten-free I**	0.96	42.53	8.88
**Gluten-free II**	0.94	18.66	3.89
**Total**	0.98	19.92	4.15

## Data Availability

The datasets generated for this study are available on request to the corresponding author, subject to demonstration of a legitimate interest.

## References

[1] Færgestad E.M., Magnus E.M., Sahlström S., Næs T. (1999). Influence of Flour Quality and Baking Process on Hearth Bread Characteristics Made Using Gentle Mixing. J. Cereal Sci..

[2] Hruskova M., Machova D. (2002). Changes of wheat flour properties during short term storage. Czech J. Food Sci.—UZPI.

[3] Psimouli V., Oreopoulou V. (2012). The effect of alternative sweeteners on batter rheology and cake properties. J. Sci. Food Agric..

[4] Alifakı Y.Ö., Şakıyan Ö. (2017). Dielectric properties, optimum formulation and microwave baking conditions of chickpea cakes. J. Food Sci. Technol..

[5] Yildiz E., Guner S., Sumnu G., Sahin S., Oztop M.H. (2018). Monitoring the Effects of Ingredients and Baking Methods on Quality of Gluten-Free Cakes by Time-Domain (TD) NMR Relaxometry. Food Bioprocess Technol..

[6] Conforti F.D. (2014). Cake Manufacture. Bakery Products Science and Technology.

[7] Wilderjans E., Luyts A., Brijs K., Delcour J.A. (2013). Ingredient functionality in batter type cake making. Trends Food Sci. Technol..

[8] Campbell G. (1999). Creation and characterisation of aerated food products. Trends Food Sci. Technol..

[9] Salazar J., Turó A., Chávez J.A., García M.J. (2004). Ultrasonic inspection of batters for on-line process monitoring. Ultrasonics.

[10] Bowler A.L., Bakalis S., Watson N.J. (2020). A review of in-line and on-line measurement techniques to monitor industrial mixing processes. Chem. Eng. Res. Des..

[11] Layton R.A., Murray W.R., Garbini J.L. (1996). The feasibility of controlling power for efficient batch mixing. Mechatronics.

[12] Layton R.A., Murray W.R., Garbini J.L. (1997). The Control of Power for Efficient Batch Mixing. Propellants Explos. Pyrotech..

[13] Kilborn R.H., Preston K.R. (1981). A dough height tracker and its potential application to the study of dough characteristics. Cereal Chem..

[14] Trinh L., Lowe T., Campbell G.M., Withers P.J., Martin P.J. (2013). Bread dough aeration dynamics during pressure step-change mixing: Studies by X-ray tomography, dough density and population balance modelling. Chem. Eng. Sci..

[15] Awad T.S., Moharram H.A., Shaltout O.E., Asker D., Youssef M.M. (2012). Applications of ultrasound in analysis, processing and quality control of food: A review. Food Res. Int..

[16] Chandrapala J., Oliver C., Kentish S., Ashokkumar M. (2012). Ultrasonics in food processing—Food quality assurance and food safety. Trends Food Sci. Technol..

[17] Khairi M.T.M., Ibrahim S., Yunus M.A.M., Faramarzi M. (2015). Contact and non-contact ultrasonic measurement in the food industry: A review. Meas. Sci. Technol..

[18] Sarkar T., Salauddin M., Kirtonia K., Pati S., Rebezov M., Khayrullin M., Panasenko S., Tretyak L., Temerbayeva M., Kapustina N. (2022). A Review on the Commonly Used Methods for Analysis of Physical Properties of Food Materials. Appl. Sci..

[19] Salimi Khorshidi A., Thandapilly S.J., Ames N. (2018). Application of low-intensity ultrasound as a rapid, cost-effective tool to wheat screening: A systematic frequency selection. J. Cereal Sci..

[20] Létang C., Piau M., Verdier C., Lefebvre L. (2001). Characterization of wheat-flour–water doughs: A new method using ultrasound. Ultrasonics.

[21] Elmehdi H.M., Page J.H., Scanlon M.G. (2004). Ultrasonic Investigation of the Effect of Mixing Under Reduced Pressure on the Mechanical Properties of Bread Dough. Cereal Chem. J..

[22] Ross K.A., Pyrak-Nolte L.J., Campanella O.H. (2004). The use of ultrasound and shear oscillatory tests to characterize the effect of mixing time on the rheological properties of dough. Food Res. Int..

[23] Elmehdi H.M., Page J.H., Scanlon M.G. (2003). Monitoring Dough Fermentation Using Acoustic Waves. Food Bioprod. Process..

[24] Skaf A., Nassar G., Lefebvre F., Nongaillard B. (2009). A new acoustic technique to monitor bread dough during the fermentation phase. J. Food Eng..

[25] Bowler A.L., Bakalis S., Watson N.J. (2020). Monitoring Mixing Processes Using Ultrasonic Sensors and Machine Learning. Sensors.

[26] Scanlon M.G., Page J.H. (2015). Probing the Properties of Dough with Low-Intensity Ultrasound. Cereal Chem. J..

[27] Strybulevych A., Leroy V., Shum A.L., Koksel H.F., Scanlon M.G., Page J.H. Use of an ultrasonic reflectance technique to examine bubble size changes in dough. IOP Conference Series: Materials Science and Engineering, Proceedings of the International Symposium on Ultrasound in the Control of Industrial Processes (UCIP 2012), Madrid, Spain, 18–20 April 2012.

[28] Allais I., Edoura-Gaena R.-B., Dufour É. (2006). Characterisation of lady finger batters and biscuits by fluorescence spectroscopy—Relation with density, color and texture. J. Food Eng..

[29] Fox P.D., Smith P.P., Sahi S.S., Yuhas D.E., Schneider S.C. (2002). Buffer rod design for measurement of specific gravity in the processing of industrial food batters. 2002 IEEE Ultrasonics Symposium, Proceedings of the An International Symposium, Forum Hotel, Munich, Germany, 8–11 October 2002.

[30] Fox P., Smith P.P., Sahi S. (2004). Ultrasound measurements to monitor the specific gravity of food batters. J. Food Eng..

[31] Gómez M., Oliete B., García-Álvarez J., Ronda F., Salazar J. (2008). Characterization of cake batters by ultrasound measurements. J. Food Eng..

[32] Colombi A., Ageeva V., Smith R.J., Clare A., Patel R., Clark M., Colquitt D., Roux P., Guenneau S., Craster R.V. (2017). Enhanced sensing and conversion of ultrasonic Rayleigh waves by elastic metasurfaces. Sci. Rep..

[33] Mayer W.G. (1965). Energy partition of ultrasonic waves at flat boundaries. Ultrasonics.

[34] Chaplain G.J., de Ponti J.M., Colombi A., Fuentes-Dominguez R., Dryburg P., Pieris D., Smith R.J., Clare A., Clark M., Craster R.V. (2020). Tailored elastic surface to body wave Umklapp conversion. Nat. Commun..

[35] Clement G.T., White P.J., Hynynen K. (2004). Enhanced ultrasound transmission through the human skull using shear mode conversion. J. Acoust. Soc. Am..

[36] Wall K. (2009). Complexity of chemical products, plants, processes and control systems. Chem. Eng. Res. Des..

[37] Gao W., Liu W., Hu Y., Wang J. (2020). Study of Ultrasonic Near-Field Region in Ultrasonic Liquid-Level Monitoring System. Micromachines.

[38] Amirkhani M., Taschin A., Cucini R., Bartolini P., Leporini D., Torre R. (2011). Polymer thermal and acoustic properties using heterodyne detected transient grating technique. J. Polym. Sci. Part B Polym. Phys..

[39] Kanatov I., Kaplun D., Butusov D., Gulvanskii V., Sinitca A. (2019). One Technique to Enhance the Resolution of Discrete Fourier Transform. Electronics.

[40] Alías F., Socoró J., Sevillano X. (2016). A Review of Physical and Perceptual Feature Extraction Techniques for Speech, Music and Environmental Sounds. Appl. Sci..

[41] Robnik-Šikonja M., Kononenko I. (2003). Theoretical and Empirical Analysis of ReliefF and RReliefF. Mach. Learn..

[42] Zhang N., Xiong J., Zhong J., Leatham K. (2018). Gaussian Process Regression Method for Classification for High-Dimensional Data with Limited Samples. Proceedings of the 8th International Conference on Information Science and Technology, ICIST 2018.

[43] Chesterton A., de Abreu D.P., Moggridge G.D., Sadd P.A., Wilson D.I. (2013). Evolution of cake batter bubble structure and rheology during planetary mixing. Food Bioprod. Process..

[44] Bowler A.L., Pound M.P., Watson N.J. (2022). A review of ultrasonic sensing and machine learning methods to monitor industrial processes. Ultrasonics.

